# Heterologous Expression of Ethanol Synthesis Pathway in Glycogen Deficient *Synechococcus elongatus* PCC 7942 Resulted in Enhanced Production of Ethanol and Exopolysaccharides

**DOI:** 10.3389/fpls.2020.00074

**Published:** 2020-02-14

**Authors:** Rajendran Velmurugan, Aran Incharoensakdi

**Affiliations:** ^1^Cyanobacterial Biotechnology Laboratory, Department of Biochemistry, Faculty of Science, Chulalongkorn University, Bangkok, Thailand; ^2^Academy of Science, Royal Society of Thailand, Bangkok, Thailand

**Keywords:** *Synechococcus elongatus*, ethanol, metabolic engineering, cofactor, exopolysaccharides

## Abstract

In this study, the *Synechococcus elongatus* PCC 7942 (hereafter *S. elongatus*) was engineered by the *glgC* knockout as well as the insertion of the *pdc*-*adh* genes from two different microorganisms. The insertion of *pdc*-*adh* genes increased the ethanol synthesis with further improvement in the productivity upon the destruction of glycogen synthesis pathway and the supplementation of cofactor. The abolition of glycogen synthesis pathway led to a considerable increase of the engineered *S. elongatus* metabolites involved in the ethanol synthesis pathway. Moreover, the studies on cofactor addition highlighted the importance of Mg^+2^, Zn^+2^, thiamine pyrophosphate, and NADP^+^ in ethanol synthesis. The yields of 3856 mg/L ethanol and 109.5 µg/10^8^ cells exopolysaccharides were obtained in the engineered *S. elongatus* using a photo-bioreactor under optimized conditions. This enhanced production in ethanol and exopolysaccharides are attributed to the flux of carbon from glycogen synthesis pathway and proper availability of essential components.

## Introduction

The energy crisis and depletion of fossil fuel necessitate the demand toward alternative fuel production. As an alternative fuel, ethanol is already alleviating the dependency on fossil fuel, and it is known to be sustainable and environmentally friendly (Mofijur et al., 2016). The ethanol production from cyanobacteria is considered as a future fuel source due to the direct fuel molecule production from atmospheric CO_2_ (Nozzi et al., 2013; Norena-Caro and Benton, 2018). Although the research on ethanol has reached up to the industrial level, there are certain factors limiting its commercialization (Su et al., 2017). In general, the engineering of ethanol synthesis pathway consisted of an insertion of two genes *pdc* and *adh* encoding pyruvate decarboxylase which converts pyruvate to acetaldehyde, and alcohol dehydrogenase which converts acetaldehyde to ethanol, respectively (Deng and Coleman, 1999). The engineering of ethanol synthesis pathway has been carried out widely in two genera, such as *Synechocystis* and *Synechococcus*, in which the genes of interest and expression systems were varied (Deng and Coleman, 1999; Gao et al., 2012). The first case of ethanol production improvement was carried out in *Synechococcus* sp. PCC 7942, which expresses PDC and ADH from *Zymomonas mobilis* under the control of the *rbcLS* promoter (Deng and Coleman, 1999). Another attempt to redirect more carbon sources to ethanol synthesis by inactivating the glycogen and PHB synthesis pathways led to an improvement of the ethanol synthesis in *Synechocystis* sp. (Gao et al., 2012; Velmurugan and Incharoensakdi, 2020). Although the engineering strategies improved the ethanol synthesis, the production process is still facing problems associated with stress tolerance, adaptability, and productivity (Pade et al., 2017). As the wild type cyanobacterial strains have certain characteristics to grow under normal growth conditions, the engineered strain to produce certain products does not grow well (Dexter and Fu, 2009). Therefore, it is important to study the growth pattern under challenging environment imposed by the incorporation of synthetic pathways. The incorporation of ethanol synthesis pathway into a new host strain may cause insufficient availability of related cofactors (Choi and Park, 2016). On the other hand, several physiological factors can decrease the product yield, such as an increased ethanol concentration, electron imbalances, and membrane transport systems (Tian et al., 2013). Higher ethanol concentration is reported to trigger stress response in cyanobacteria (Tian et al., 2013). The *Synechococcus* holds its natural traits like possessing various unique components in cyanobacteria like exopolysaccharides, glycogen, and carotenoids, and these components act as an extracellular protecting agent and intracellular electron sink, respectively (Pereira et al., 2009; Velmurugan and Incharoensakdi, 2018). As one of the strains developed in this study is defective in glycogen synthesis, the demonstration of the changes of exopolysaccharides content in response to stress can be informative.

In this study, the ethanol synthesis pathway engineered *S. elongatus* was used to analyze the ethanol production and also to investigate the changes in intracellular and extracellular concentration of biomolecules. The contribution of cofactor has been evaluated to fulfill the ethanol synthesis pathway as the pathway is new to the host strain *S. elongatus*. Besides, the exopolysaccharides content in response to physiological condition imposed by pathway engineering was determined.

## Materials and Methods

### Materials

All the standard chemicals used were purchased from Sigma-Aldrich (USA) and the nucleotide bases were purchased from Fermentas (Canada). Taq polymerase, ligase, and restriction enzymes (BamHI, BmtI, HindIII, KpnI, and NdeI) were purchased from Fermentas (Canada). The kits used for plasmid extraction and PCR purification were obtained from Geneaid Biotech Ltd. Oligonucleotide primers were designed with the help of primer3 online software, and the synthesis was performed by Pacific Science Co. Ltd, Thailand. The vectors such as pSyn_1, pGEM-T easy, and pUC4K were standard commercial products.

### Strains and Cultivation Conditions

*Synechococcus elongatus* PCC 7942 (Pasteur Institute, France) was propagated on BG-11 agar medium (Rippka et al., 1979). The wild type and engineered strains were cultivated in 250 ml Erlenmeyer flasks containing 100 ml BG-11 medium (pH 7.5) under continuous illumination of 50 μE/m^2^/s at 28 ± 1°C with atmospheric CO_2_ supplementation. The commercial microorganisms *Escherichia coli* DH5α and *Saccharomyces cerevisiae* (MTCC-170) were cultivated in LB medium and, yeast extract peptone and dextrose medium (YEPD), respectively.

### Plasmid Construction

**Figure 1:**
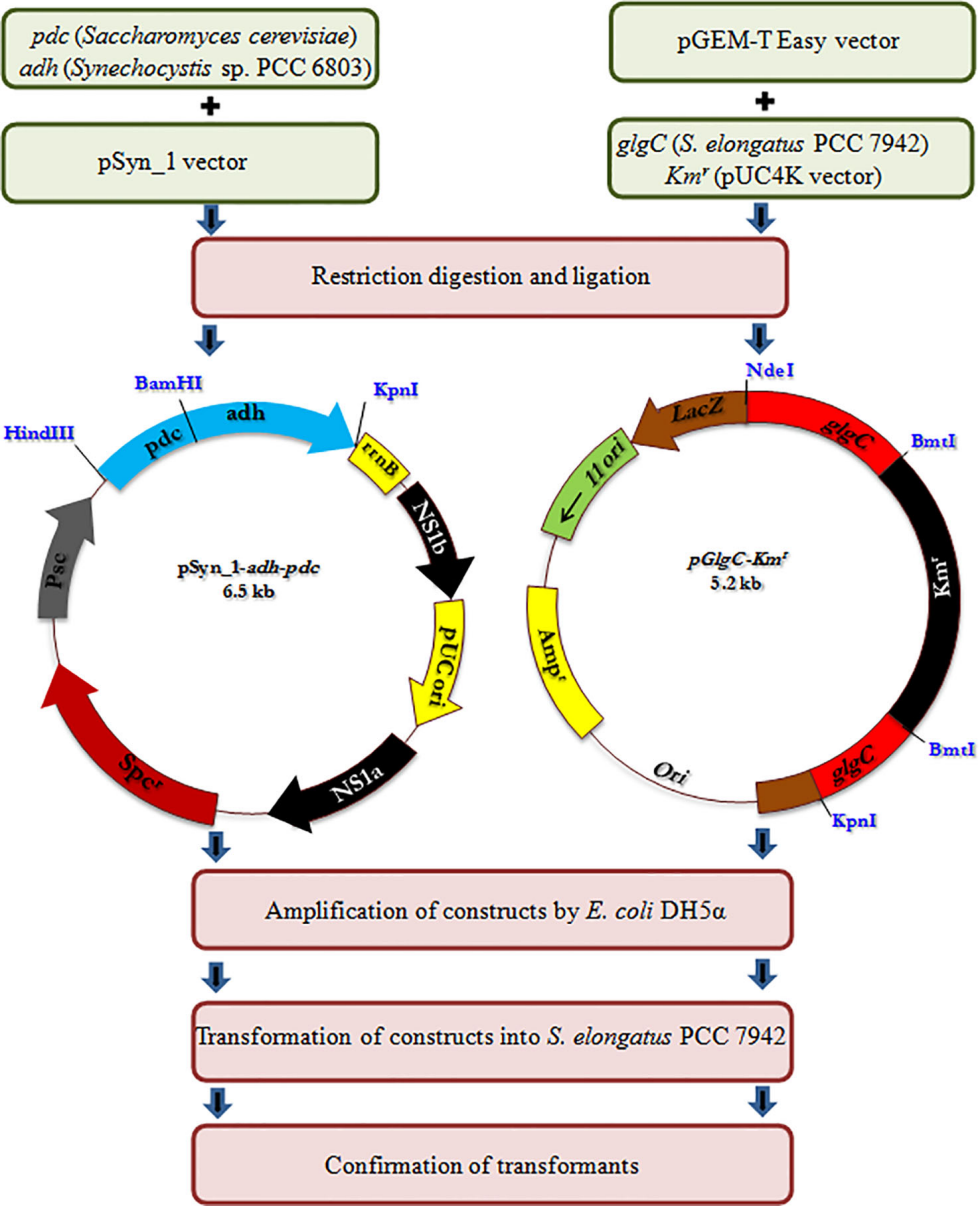
Schematic representation of engineering of *S. elongatus*.

All the primers used and the strains constructed are presented in **Table 1** and **Figure 1**, respectively. The expression vector pAPX was constructed by the insertion of NADP^+^ dependent alcohol dehydrogenase (*adh*: slr0942) into pSyn_1 vector under the control of *Psc* promoter. The gene *adh* was amplified with respective primers (0942F and 0942R) using the genomic DNA of *Synechocystis* as a template and the gene *pdc* was amplified with respective primers (*PdcF* and *PdcR*) using the genomic DNA of *S. cerevisiae* as a template (**Figure 1**). Plasmid pGK was constructed by inserting 1.3 kb ADP-glucose pyrophosphorylase (*glgC*) gene into pGEM-T easy vector. The *glgC* was amplified with respective primers (0603F and 0603R) using the genomic DNA of *S. elongatus* as a template. The inactivation of *glgC* was carried out by inserting a 0.92 kb kanamycin resistance cassette from pUC4K vector into BmtI site of pGK resulting in plasmid with kanamycin resistance.

**Table 1:** Primers used for engineering of *S. elongatus* PCC 7942.

Gene	Primers	Oligonucleotide	PCR product length (bp)
*adh*	0942F	GTGGATCCGTGCAGAGTTTCAATAGG	990
	0942R	CGGGTACCTTAAATTTCATCCCATAGG	
*pdc*	PdcF	GCGGAAGCTTATGTCTGAAATTACTTTG	1698
	PdcR	GCGGATCCTTATTGCTTAGCGTTGGT	
*glgC*	0603F	TGGTACCGTGAAAAACGTGCTGGCGAT	1299
	0603R	GTCATATGTTAGATCACCGTGTTGTCGGG	
Km	KmF	GCAAGCTAGCAAGCCACGTTGTGTCTCA	932
	KmR	GCCAGCTAGCGATTAGAAAAACTCATCG	
Adh-RT-PCR	Adh-RTF	AACTTTGCAGGATTTGGGTCTA	297
	Adh-RTR	AGCAAGTCTGATTGTTGGAGGTA	
Pdc- RT-PCR	Pdc-RTF	CCAGCTTTCGTCACCCCAAT	265
	Pdc-RTR	CGAATTTCATTTGGACACCTGG	
16s	16s-RTF	CTTCGCGTTGCATCGAATTAAACCAC	368
	16s-RTR	GCGTGGGGCTCAACCTCATAC	
NSI	NS1F	GGCAGCTTGGAAGGGCG	1568
	NS1R	GGCGTTGCCAATATCAAGATTGC	
Promoter	pSyPF	CGGTCTGATCTTAGCGG	Not applicable

### Construction of Engineered Strains

*Escherichia coli* DH5α strain was used for routine propagation of all plasmid constructs and was cultivated in LB medium containing respective antibiotics at appropriate concentration. The transformation of plasmid constructs into *E. coli* DH5α was performed by heat shock transformation and the amplified plasmids were extracted after the cultivation for overnight. The engineered *S. elongatus* was obtained by natural transformation. Briefly, *S. elongatus* was grown in BG-11 medium for 2 to 3 days until the cell density reached OD_730_ = 0.3. Cells were harvested by centrifugation and resuspended in fresh BG-11 medium (100 µL). The transformation was carried out by adding 5µL of appropriate plasmid DNA into the cell suspension. The mixture was incubated at 27°C under low light for 6 h before spreading on a plate containing appropriate antibiotics for natural transformation. After about 2 weeks of incubation, the single colonies were isolated and subcultured for at least seven generations. After full segregation was achieved, engineered strains were verified by forward and reverse primers of NS1 (NS1F and NS1R), *glgC* (0603F and 0603R), and antibiotic cassette (KmF and KmR), respectively (**Figure S1**). The engineered strains were maintained in BG-11 medium containing appropriate antibiotics such as ampicillin (30 µg/ml), kanamycin (50 µg/ml), and spectinomycin (30 µg/ml) (Kanwal et al., 2015).

### Characterization of Wild-Type and Engineered Strains

The wild type and engineered *S. elongatus* were cultured in 250 ml Erlenmeyer flasks containing 100 ml BG11 medium (pH 7.5) with a continuous illumination of 100 μE/m^2^/s at 28 ± 1°C and atmospheric CO_2_ was supplemented. The initial cell density was 4 × 10^7^ cells/ml. The optimization of cofactor concentration such as MgSO_4_.7H_2_O (0, 200, 400, 600, and 800 µM), ZnSO_4_.7H_2_O (0, 1, 2, 3, 4 µM), TPP (0, 50, 100, 150, and 200) and NADP^+^ (0, 50, 100, 150, and 200) concentrations were optimized in 250-ml flask experiment as outlined above. The individual effect of cofactors on growth, exopolysaccharides, and ethanol content was analyzed in 5 L photo-bioreactor with the atmospheric CO_2_ supplementation at the flow rate of 200 ml/min. Cells were harvested after 20 days of growth by centrifugation at 5,000*g* for 10 min at room temperature. To estimate the intracellular metabolites, cells were vigorously mixed with 500 μl of 70% methanol by vortex mixer. The mixture was incubated for 2 h at room temperature and then centrifuged at 6,000*g* for 10 min at 4°C. The supernatant was collected and dried in a vacuum evaporator at 40°C. The pellet left after drying was dissolved and mixed thoroughly in 250 μl of water and 50 μl of chloroform followed by centrifugation at 6,000*g* for 10 min. The uppermost water phase (200 μl) was collected, and filtered through a 0.45 μm Millipore filter before the analysis by high performance liquid chromatography (HPLC).

To estimate the exopolysaccharides content, the cell suspension obtained from 50-ml culture was made up to 10 ml using Milli-Q water (Millipore, USA) and vortexed five times for 30 s with 1 min intervals. The samples were centrifuged at 15,000*g* for 20 min under room temperature, and the supernatant was concentrated approximately 10 fold by evaporation at 60°C for 8 to 12 h. The exopolysaccharides in the concentrated liquid were precipitated by gradual addition of 3 volumes of cold ethanol and kept overnight at 4°C. After evaporation, the precipitate was washed with cold absolute ethanol, followed by centrifugation. The gel-like pellet was dialyzed against 5 volumes of MilliQ water at room temperature for 12 h. The samples were dissolved in HCl (2M final) and autoclaved at 110°C for 10 min. The concentrations of glucose, galactose, xylose, mannose, arabinose, and uronic acid were determined by HPLC (Panoff et al., 1998).

### Transcript Analysis by RT-PCR

Total RNA was extracted by using the TRI Reagent (Molecular Research Center). After DNase (Fermentas) treatment, single-stranded cDNA was synthesized from 1 μg of total RNA with the SuperScript™ III First-Strand Synthesis Kit (Invitrogen) according to the manufacturer’s instruction. RT-PCR using cDNA of the *adh*, *pdc*, and *16s* as a reference gene was performed using forward and reverse primers as described in **Table 1**. The PCR conditions consisted of: denaturation at 94°C for 5 min, followed by 20 cycles of 94°C for 30 s, annealing temperature of 55°C for 1 min and 72°C for 20 s, and then a final extension at 72°C for 3 min. The PCR product was analyzed using a 1.0% (w/v) agarose gel electrophoresis system (Kanwal et al., 2014).

### Analytical Methods

Intracellular pigments of *S. elongatus* cell suspension were extracted by dimethylformamide. Chlorophyll *a* and carotenoid contents were determined according to the methods of Moran (1982) and Chamovitz et al. (1993), respectively. A Clark-type oxygen electrode was employed for oxygen evolution measurement (YSI 5300A, YSI Inc., USA) at 30°C (Jantaro et al., 2005). PHB contents were determined using HPLC (Shimadzu, Japan) equipped with InertSustain 3-µm C18 column (GL Sciences, Japan) and UV/Vis detector as described by Monshupanee and Incharoensakdi (2014). The estimation of glycogen was performed as previously described (Velmurugan and Incharoensakdi, 2016). The sugar (glucose, galactose, xylose, mannose, and arabinose) and ethanol contents were quantified using HPLC system equipped with refractive index detector (RID 10A, Shimadzu, Japan). Metabolic intermediates such as acetate, pyruvate, succinate, and uronic acid were quantified using HPLC equipped with UV/Vis detector (SPD-20A, Shimadzu, Japan). The components were separated in Phenomenex, Rezex ROA-Organic acid column (150 × 7.8 mm) with 5 mM H_2_SO_4_ as a mobile phase at a flow rate of 0.6 ml/min (Velmurugan and Incharoensakdi, 2017).

### Enzyme Characterization

The intracellular protein was extracted from the cells after 10 and 20 days of cultivation. Cells were harvested from 50-ml liquid culture by centrifugation at 6,000*g* for 10 min and resuspended in 3 ml of 30 mM Tris-HCl, pH 8.0. The resuspended cells were lysed by ultrasonic treatment for 30 s with 60% power input and were repeated three times in a pre-chilled water bath. The lysate was centrifuged at 6000*g* for 5 min, and the supernatant was used for protein determination, ADH and PDC assays.

The protein concentration was determined by Bradford method using BSA as a standard protein (Bradford, 1976). Activities of ADH and PDC were measured by monitoring the increase and decrease in absorbance at 340 nm with utilization of 200 µM NADP^+^ and NADPH, respectively. Briefly, the ADH activity was measured by adding the enzyme extract in 30 mM Tris (pH 8.0) buffer containing 200 µm NADP^+^ and ethanol. The PDC activity was measured in 100 mM Tris (pH 7.5) buffer containing 200 µm NADPH, 0.1 mM MgCl_2_, 0.1 mM thiamine pyrophosphate, and 10 mM pyruvate (Gao et al., 2012).

### Statistical Analysis

The experiments were performed with three biological replicates, and the average values are reported. The average standard deviation values were calculated using the respective functions (AVERAGE, STDEV) available in Microsoft Excel. The significance of the results were analysed by two-tailed Student’s t-test.

## Results

### Growth of Wild Type and Engineered Strain

**Figure 2:**
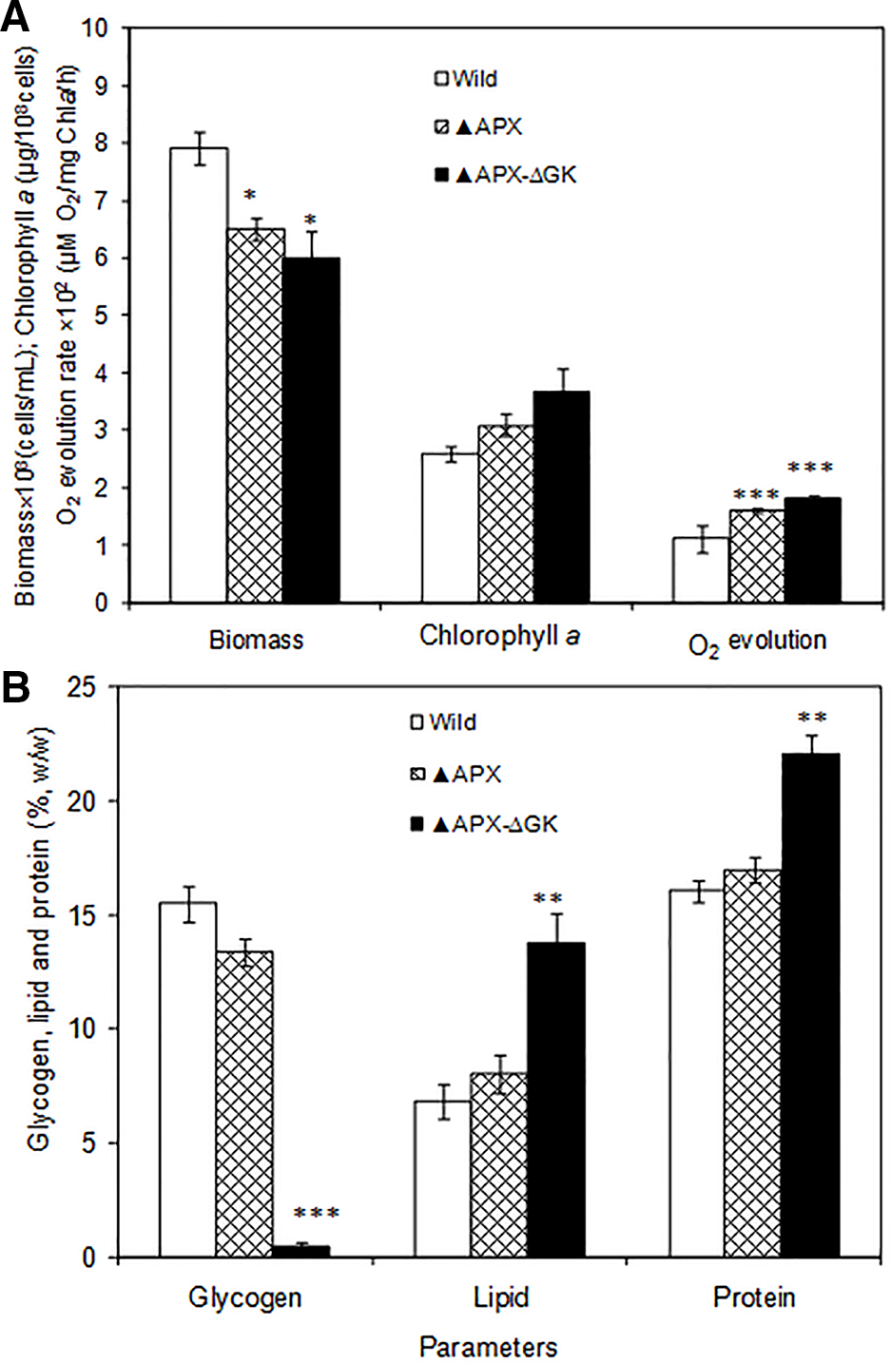
**(A)** Biomass and chlorophyll *a* content and oxygen evolution rate and **(B)** glycogen, lipid and protein contents in wild type and engineered *S. elongatus* (conditions: 20 days; BG-11 medium). The values are mean ± standard deviation (n = 3), statistical significance (compared to wild type) are indicated by * for *p* < 0.05; ** for *p* < 0.01; *** for *p* < 0.001.

The growth pattern of wild type, *adh-pdc* overexpressing strain (▲APX) and *adh-pdc* overexpressing strain containing *glgC* knockout (▲APX-ΔGK) under photosynthetic growth condition is shown in **Figure 2A**. *The* two engineered strains (▲APX and ▲APX-ΔGK) had lower growth than the wild type strain, whereas the *adh-pdc* overexpressing strain containing *glgC* knockout (▲APX-ΔGK) showed the lowest growth of 6.04 × 10^8^ cells/ml, suggesting that the glycogen plays an important role in the cell growth. In addition, the culture broth of the *adh-pdc* overexpressing strain containing *glgC* knockout (▲APX-ΔGK) showed slightly yellowish color while the wild type culture stayed greenish at least until 20 days. The insertion of ethanol synthesis pathway improved the chlorophyll *a* content and the disruption of glycogen synthesis further improved the chlorophyll *a* content of 3.50 µg/10^8^ cells (**Figure 2A**). The increase in chlorophyll *a* content was observed concomitant with the decrease in glycogen content of both *adh-pdc* overexpressing strain (▲APX) and *adh-pdc* overexpressing strain containing *glgC* knockout (APX-ΔGK) (**Figure 2B**). The oxygen evolution rate was also increased in engineered strains in a similar manner to the increase of chlorophyll *a* with the highest rate of 1.8 × 10^2^ µmol O_2_/mg chl*a*·h observed in the overexpressing strain containing glycogen synthesis knockout (**Figure 2A**).The wild type strain contained 15.5% glycogen content which was slightly reduced to 13.4% in the strain engineered for enhanced ethanol production (▲APX) and drastically reduced to 0.5% in overexpressing strain containing glycogen synthesis knockout (▲APX -ΔGK) (**Figure 2B**). On the other hand, the lipid and protein contents were increased to 13.8 and 22% w/w respectively in ▲APX -ΔGK strain (**Figure 2B**).

### Metabolic Changes in Engineered Strains

**Figure 3:**
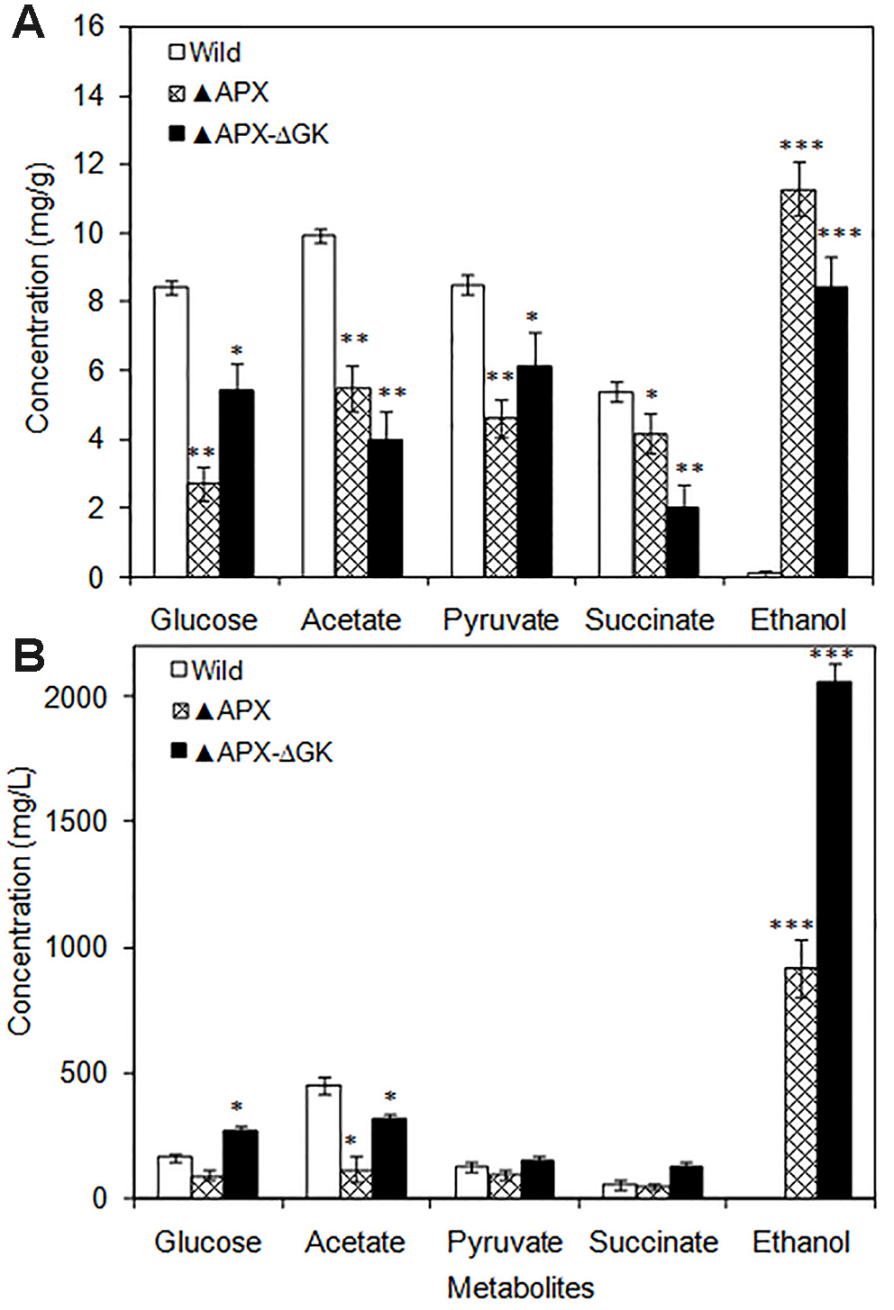
**(A)** Intracellular and **(B)** extracellular metabolites of wild type and engineered *S. elongatus* (conditions: 20 days; BG-11 medium). Values are mean ± standard deviation (n = 3), statistical significant values (compared to wild type) are indicated by * for *p* < 0.05; ** for *p* < 0.01; *** for *p* < 0.001.

The intracellular glucose, acetate, pyruvate, and succinate contents were drastically decreased in engineered strains as compared to those in the wild type (**Figure 3A**). Notably, the intracellular ethanol content was remarkably increased in the overexpressing strains. When comparing the *adh-pdc* overexpressing strain (▲APX) with *adh-pdc* overexpressing strain containing *glgC* knockout (▲APX -ΔGK), the extracellular concentrations of glucose, acetate, pyruvate, and succinate were increased in glycogen synthesis knockout strain indicating the excessive amount of these three metabolites being excreted out when their intracellular contents exceeded the limit (**Figure 3B**). Remarkably, the ethanol production is highly improved from 919.7 mg/L to 2059.7 mg/L by the *glgC* knockout in *S. elongatus* (**Figure 3B**).

### Expression Levels of Engineered Strains

**Figure 4:**
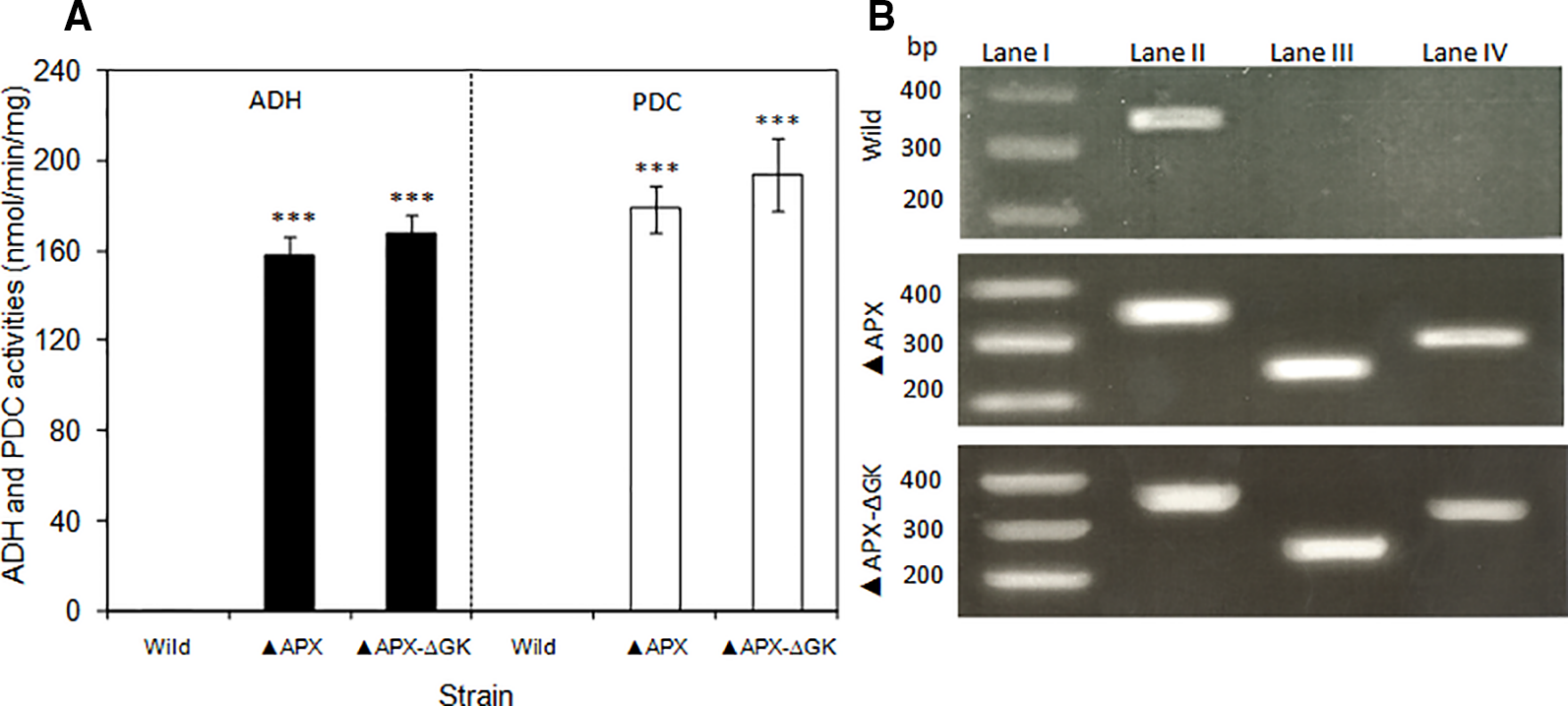
**(A)** Activities of pyruvate decarboxylase and alcohol dehydrogenase of wild type and engineered *S. elongatus* and **(B)** electrophoresis of RT-PCR products of wild type and engineered *S. elongatus*. Lane I: gene marker, lane II: 16s, lane III: *pdc*, lane IV: *adh*. (conditions: BG-11 medium; 3 days for RT-PCR, 20 days for enzyme activity). Values are mean ± standard deviation (n = 3), statistical significant values (compared to wild type) are indicated by *** for *p* < 0.001.

The expression level of *adh* and *pdc* genes were quantified by analyzing their enzyme activities and the *adh* and *pdc* transcripts of total RNA extracted from *S. elongatus* cells (wild type, *adh-pdc* overexpressing; ▲APX, and *adh-pdc* overexpressing strain containing *glgC* knockout; ▲APX-ΔGK) (**Figures 4A, B**). Since multiple copies of chromosomes exist in S. *elongatus*, the *16s* gene was chosen as a reference gene. As shown in **Figure 4B**, the transcripts of both *adh* and *pdc* were not detected in the wild type while they were present in high amounts in the engineered strains, indicating the successful segregation of respective genes in the *S. elongatus*. The transcript level of *adh* was not much different for both *adh-pdc* overexpressing strain (▲APX) and *adh-pdc* overexpressing strain containing *glgC* knockout (▲APX-ΔGK), whereas the latter showed a 1.5-fold increase in *pdc* transcript level. The maximum transcript level of 0.55 and 0.60 were observed for *adh* and *pdc* respectively in *adh-pdc* overexpressing strain containing *glgC* knockout (▲APX-ΔGK). Subsequently, the activities of ADH and PDC in the wild type, ▲APX and ▲APX-ΔGK strains were examined (**Figure 4A**). In accord with the transcript levels, the enzyme activities were not observed in the wild-type, whereas the *adh-pdc* overexpressing strain (▲APX) and *adh-pdc* overexpressing strain containing *glgC* knockout (▲APX-ΔGK) had considerable enzyme activities, in which the *adh-pdc* overexpressing strain containing *glgC* knockout (▲APX-ΔGK) produced the maximum ADH and PDC activities of 168 and 194 nmol/min/mg respectively.

### Contribution of Cofactors in Ethanol Synthesis

**Figure 5:**
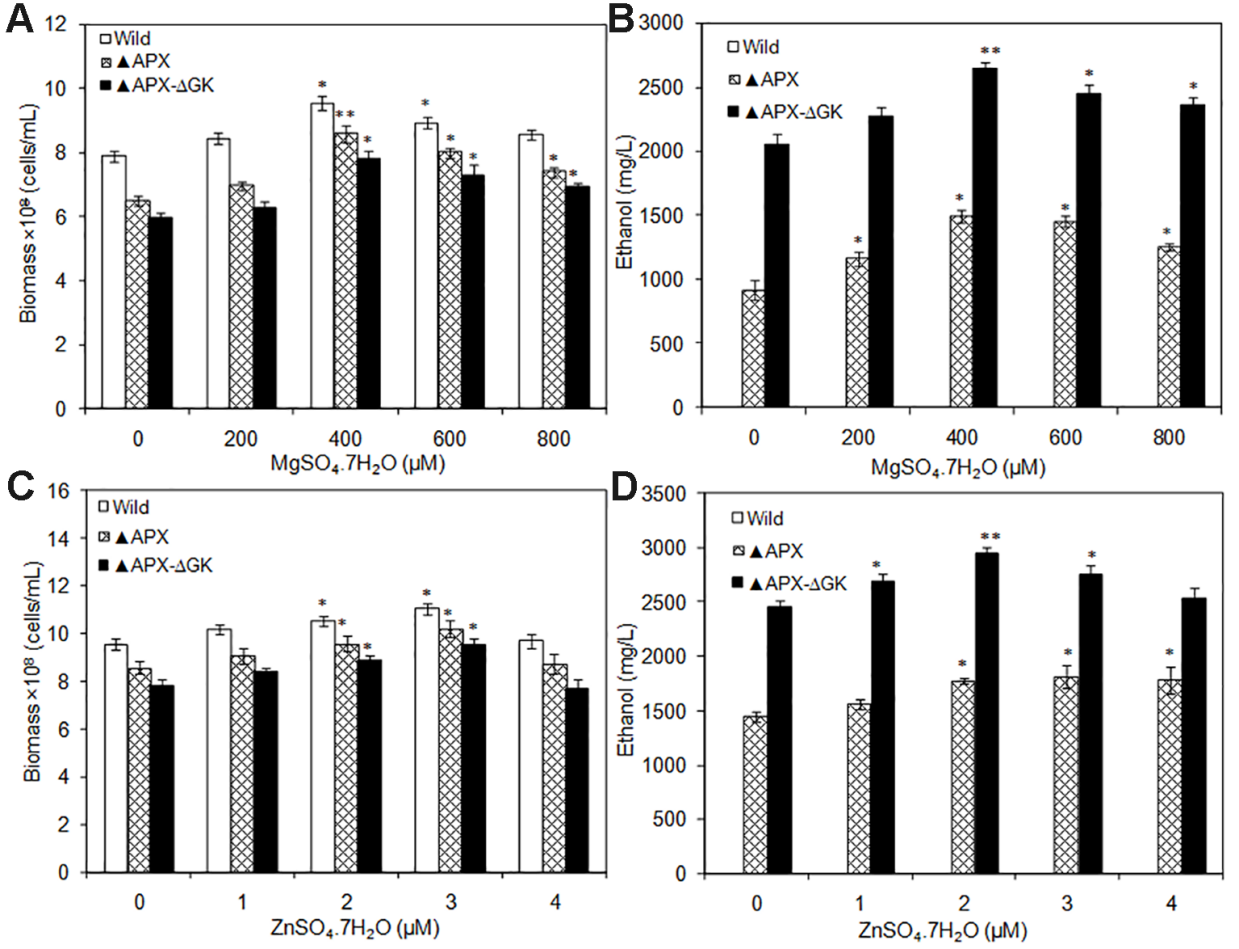
**(A**, **B)** Influence of MgSO_4_.7H_2_O addition on biomass and ethanol production (conditions: 20 days; BG-11 medium) and **(C**, **D)** Influence of ZnSO_4_.7H_2_O addition on biomass and ethanol production (conditions: 20 days; BG-11 medium supplemented with 400 µM MgSO_4_.7H_2_O). Values are mean ± standard deviation (n = 3), statistical significant values (compared to no supplementation) are indicated by * for *p* < 0.05; ** for *p* < 0.01.

To analyze the actual requirement of Mg^+2^ and Zn^+2^ for cell growth and ethanol production, the MgSO_4_·7H_2_O and ZnSO_4_·7H_2_O were supplemented by varying their concentrations. Under all Mg^+2^ concentrations tested, the growth of the wild type was highest followed by that of *adh-pdc* overexpressing strain (▲APX) and *adh-pdc* overexpressing strain containing *glgC* knockout (▲APX-ΔGK) respectively (**Figure 5A**). Nevertheless, the growth of all three strains was improved with an increase in the concentration of Mg^+2^ (**Figure 5A**). At 400 µM of Mg^+2^, the *adh-pdc* overexpressing strain containing *glgC* knockout (▲APX-ΔGK) showed maximum ethanol concentration of 2643 mg/L (**Figure 5B**) with the cell count of 7.82 × 10^8^ cells/ml (**Figure 5A**). Similar to the effect by Mg^+2^, the Zn^+2^ also increased ethanol production upon an increase in the concentration of Zn^+2^ and the maximum ethanol concentration of 2943 mg/L was observed at 2 µM of Zn^+2^ with the cell count of 8.89 × 10^8^ cells/ml in *adh-pdc* overexpressing strain containing *glgC* knockout (▲APX-ΔGK) (**Figures 5C, D**). The optimization of metals (MgSO_4_·7H_2_O and ZnSO_4_·7H_2_O) concentrations together improved the ethanol and biomass production up to 1.43 and 1.48 folds, respectively, compared to that with normal BG-11 medium without metal supplementation.

**Figure 6:**
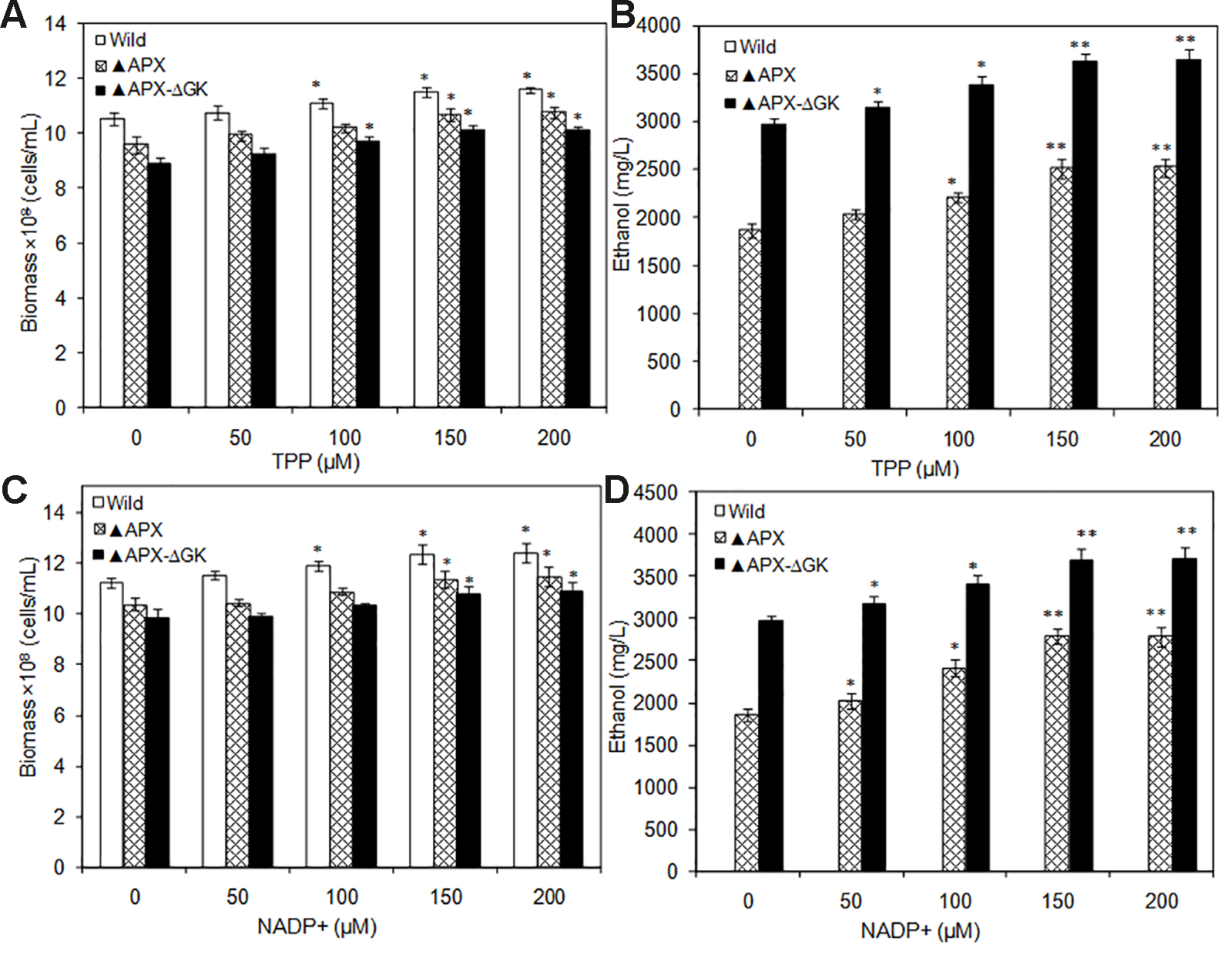
**(A**, **B)** Influence of TPP addition on biomass and ethanol production (conditions: 20 days; BG-11 medium supplemented with 400 µM MgSO_4_.7H_2_O; 2 µM ZnSO_4_.7H_2_O), **(C**, **D)** Influence of NADP^+^ addition on biomass and ethanol production (conditions: 20 days; BG-11 medium supplemented with 400 µM MgSO_4_.7H_2_O; 2 µM ZnSO_4_.7H_2_O and 150 µM TPP). Values are mean ± standard deviation (n = 3), statistical significant values (compared to no supplementation) are indicated by * for *p* < 0.05; ** for *p* < 0.01.

The TPP and NADP^+^ act as co-factors for PDC and ADH, respectively. Moreover, TPP is an essential cofactor for various enzymes such as transketolase, α-ketoglutarate dehydrogenase, pyruvate dehydrogenase, and α-keto acid dehydrogenase. The catalytic reactions mediated by these enzymes are the major source of ATP, NADPH, and ribose-5-phosphate. As a result of an increase in overall cellular energy, the biomass contents of the wild type and the engineered strains were increased upon TPP addition. The wild type strain had increased biomass with the supplementation of TPP, while the ethanol synthesis pathway engineered strains showed reduced biomass, even in the absence of glycogen synthesis (**Figure 6A**). The maximum biomass of 11.4 × 10^8^ cells/ml was observed in the wild type at 150 µM TPP. The ethanol production was also increased by TPP significantly and reached the maximum at 150 µM TPP (**Figure 6B**). On the other hand, the supplementation of NADP^+^ had beneficial effect on *S. elongatus* growth, as it is a major source of ATP synthesis (**Figure 6C**). The maximum ethanol concentration of 3857 mg/L was observed at 2 µM NADP^+^, which is 1.3-fold and 1.9-fold higher than that in metal supplemented medium and normal BG-11 medium, respectively (**Figure 6D**).

### Exopolysaccharides Accumulation in Response to Pathway Engineering and Cofactor Supplementation

**Figure 7:**
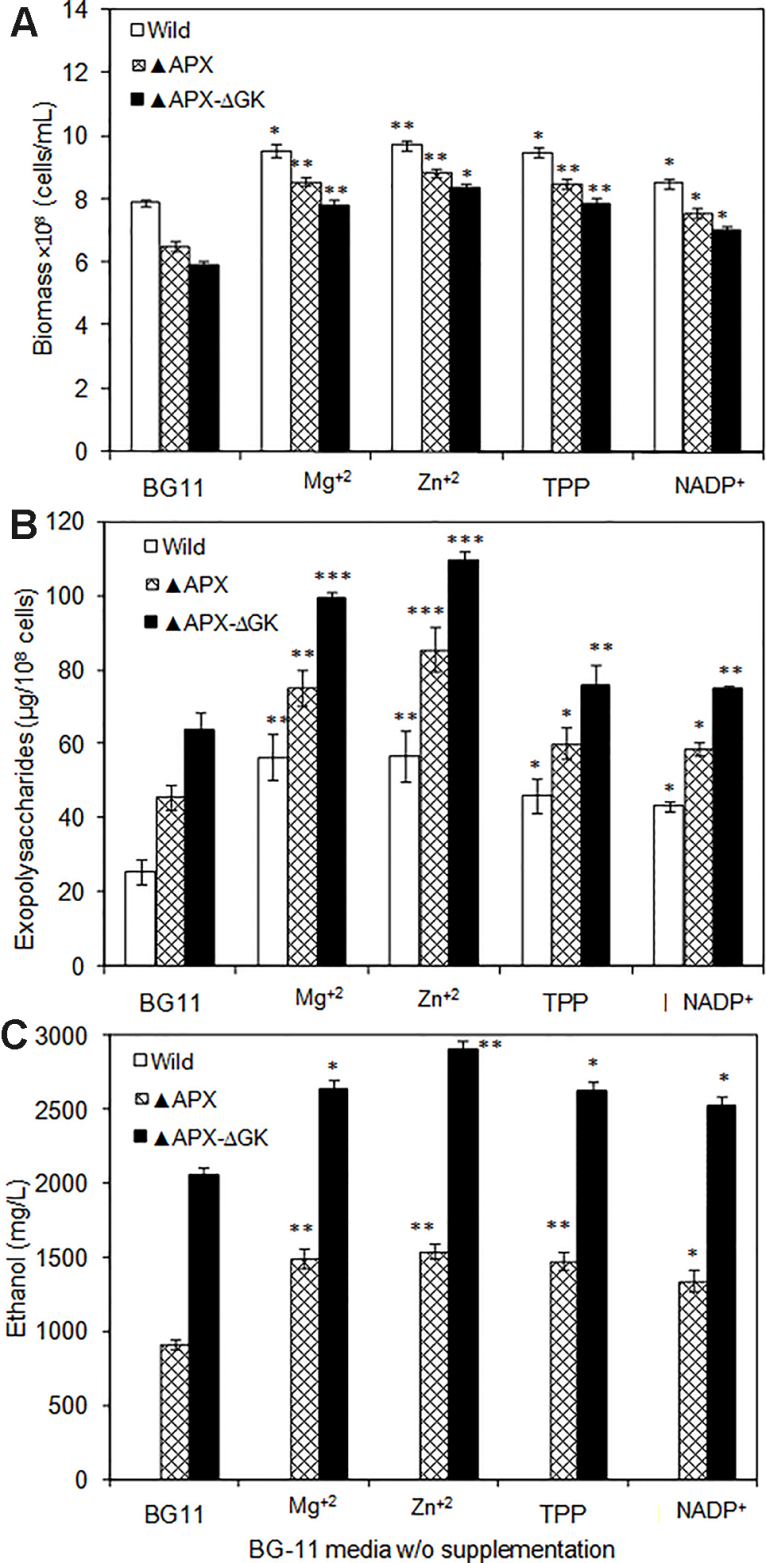
**(A)** Biomass, **(B)** exopolysaccharides, and **(C)** ethanol production in wild type and engineered *S. elongatus* under various conditions (conditions: 20 days; BG-11 medium or BG-11 medium supplemented with 400 µM MgSO_4_·7H_2_O or 2 µM ZnSO_4_·7H_2_O or 150 µM TPP or 150 µM NADP^+^). Values are mean ± standard deviation (n = 3), statistical significant values (compared to no supplementation) are indicated by * for *p* < 0.05; ** for *p* < 0.01; *** for *p* < 0.001.

As can be seen in **Figures 7A–C**, the biomass, exopolysaccharides, and ethanol concentration was varied upon cofactor supplementation in BG11 medium. The production pattern of exopolysaccharides and ethanol was very similar to each other. Although the wild type produced the exopolysaccharides at detectable level (25.5 µg/10^8^ cells), the concentration of exopolysaccharides in engineered strain ▲APX was considerably improved (45.7 µg/10^8^ cells). On the other hand, the *adh-pdc* overexpressing strain containing *glgC* knockout (▲APX-ΔGK) improved the exopolysaccharides content further to 63.9 µg/10^8^ cells (**Figure 7B**). The major components of exopolysaccharides were analyzed to measure the alternative sugar monomer produced in place of the glycogen (**Table 2**). As expected, the contents of glucose, galactose, xylose, and mannose in exopolysaccharides were increased upon engineering the strain. When comparing the *adh-pdc* overexpressing strain (▲APX) with *adh-pdc* overexpressing strain containing *glgC* knockout (▲APX-ΔGK), the concentration of these sugars was higher in glycogen synthesis knockout strain which confirm the redirection of carbon sources from glycogen synthesis pathway to exopolysaccharides synthesis (**Table 2**). The contents were increased further upon the addition of co-factors. The maximum exopolysaccharides content of 109.5 µg/10^8^ cells was observed in Zn^+2^ supplemented *adh-pdc* overexpressing strain containing *glgC* knockout (▲APX-ΔGK).

**Table 2:** Composition of exopolysaccharides in wild type and engineered strains.

Strain	Glucose (µg/ml)	Galactose (µg/ml)	Xylose (µg/ml)	Mannose (µg/ml)	Arabinose (µg/ml)	Uronic acid (µg/ml)
BG-11						
Wild type	30 ± 1.6	42 ± 1.5	20 ± 2.2	27 ± 0.6	54 ± 2.8	29 ± 0.3
▲APX	58 ± 2.3***	54 ± 6.1***	37 ± 3.3***	40 ± 2.5***	65 ± 1.3***	43 ± 1.5***
▲APX-ΔGK	87 ± 6.4***	81 ± 2.1***	58 ± 0.9***	47 ± 2.9***	76 ± 2.8***	30 ± 1.0
Mg^+2^						
Wild type	103 ± 3.8	109 ± 3.4	59 ± 1.7	76 ± 4.9	106 ± 3.7	81 ± 3.5
▲APX	105 ± 4.7	104 ± 2.7	72 ± 2.6***	114 ± 6.1***	135 ± 5.1***	109 ± 4.8***
▲APX-ΔGK	142 ± 2.4***	123 ± 1.1**	93 ± 4.1***	125 ± 4.0***	187 ± 4.6***	104 ± 2.6***
Zn^+2^						
Wild type	85 ± 2.5	78 ± 5.2	56 ± 3.3	71 ± 3.5	112 ± 3.9	83 ± 4.1
▲APX	106 ± 6.7***	113 ± 3.2***	93 ± 4.1***	119 ± 5.0***	122 ± 3.8**	95 ± 3.5^*^
▲APX-ΔGK	136 ± 4.3***	146 ± 4.8***	106 ± 4.6***	131 ± 4.1***	149 ± 4.2***	101 ± 4.0**
TPP						
Wild type	77 ± 3.9	84 ± 2.5	50 ± 1.3	68 ± 2.9	121 ± 5.7	76 ± 3.6
▲APX	97 ± 4.7**	100 ± 4.1**	87 ± 2.8***	88 ± 3.3**	119 ± 3.8	76 ± 2.7
▲APX-ΔGK	145 ± 2.2***	143 ± 5.9***	115 ± 3.9***	85 ± 3.4**	135 ± 3.3^*^	58 ± 1.4^*^
NADP^+^						
Wild type	71 ± 4.1	81 ± 3.6	51 ± 1.4	62 ± 3.8	95 ± 5.3	60 ± 1.1
▲APX	90 ± 3.0**	93 ± 2.4^*^	67 ± 4.6^*^	83 ± 1.4***	104 ± 4.1	82 ± 2.9**
▲APX-ΔGK	128 ± 5.8***	126 ± 4.0***	106 ± 5.1***	88 ± 2.1***	121 ± 5.5**	59 ± 0.9

Values are mean ± standard deviation (n = 3), statistical significant values (compared to wild type) are indicated by * for p < 0.05; ** for p < 0.01; *** for p < 0.001.

## Discussion

### Influence of Pathway Engineering in *S. elongatus*

The reduction in cell growth upon engineering of ethanol synthesis pathway in *S. elongatus* (▲APX) with amore reduction of growth when further engineered with glycogen synthesis knockout (▲APX-ΔGK) suggested the negative impact on cell growth upon engineering of the strain; moreover, it also highlighted the important role of glycogen in the cell growth (**Figure 2A**). The improvement in lipid and protein contents in the engineered strains occurred in concomitant with the decrease of glycogen content, suggesting the possible break down of glycogen as well as the re-direction of carbon flow towards the synthesis of lipid and protein rather than toward glycogen synthesis. The huge storage of carbon sources like glycogen in glycogen synthesis knockout strain makes it possible to provide the carbon substrate in the ethanol synthesis pathway, even though the glycogen synthesis pathway and ethanol synthesis pathway seem unlikely to be competitive. The increase in acetate (source of lipid biosynthesis), succinate (TCA cycle intermediate), pyruvate (source of ethanol synthesis pathway) concentration in *adh-pdc* overexpressing strain containing *glgC* knockout (▲APX-ΔGK) also confirms the redirection of carbon sources towards ethanol synthesis (**Figure 3A**). On the other hand, the extracellular concentrations of these metabolites were also higher in *adh-pdc* overexpressing strain (▲APX) compared to that in *adh-pdc* overexpressing strain containing *glgC* knockout (▲APX-ΔGK), indicating the possible excretion of excess carbon sources by *S. elongatus* (**Figure 3B**). The excretion of succinate, pyruvate, and acetate has already been previously reported for *S. elongatus* (Hickman et al., 2013). The improvement in extracellular glucose, pyruvate, and succinate concentrations indicated the overproduction due to the lack of glycogen synthesis in the *glgC* knockout strain.

### Cofactor Supplementation Improves Ethanol Production

*S. elongatus* has been engineered to produce ethanol, as it does not carry an effective natural ethanol synthesis pathway. The production of ethanol from *S. elongatus* has been initiated by the incorporation of *pdc* and *adh* from *Zymomonas mobilis* and produced maximum ethanol concentration of 5 mM (≈0.23 g/L) (Deng and Coleman, 1999). Recently, Kopka et al. (2017) engineered *pdc* from *Zymomonas mobilis* and *adh* from *Synechocystis* sp. PCC 6803 in *Synechococcus* sp. PCC 7002 and observed the production of 0.25% (v/v) ethanol. In general, Mg^+2^ and Zn^+2^ are the cofactors for PDC and ADH, respectively. The incorporation of respective genes (*pdc* and *adh*) into a new host might disturb the availability of cofactors for enzymes in ethanol synthesis pathway. Noticeably, both biomass and ethanol production were increased upon MgSO_4_·7H_2_O and ZnSO_4_·7H_2_O supplementation up to a certain concentration, which signify the requirement of more cofactors for ethanol synthesis pathway engineered strain. The results clearly indicate that both cofactors are important requirements of ethanol synthesis pathway, in which the Mg^+2^ is more effective for cell growth and ethanol production. It is noted that the wild type showed no ethanol production either with or without Mg^+2^ and Zn^+2^ addition (**Figures 5B, D**).

Similarly, the co-factors such as TPP and NADP^+^ supplementation improved the biomass and ethanol production. This improvement is due to the contribution of those cofactors for ethanol production as well as other cellular functions. Recent computational modeling studies demonstrated that the intracellular ATP and NADPH concentrations could enhance the biofuel production (Shabestary and Hudson, 2016). In particular, the decrease in ATP to NADPH ratio was predicted to enhance the ethanol production (Shabestary and Hudson, 2016). According to this hypothesis, the increase in NADPH concentration can automatically decrease the ATP to NADPH ratio, which can further increase the ethanol production. Choi and Park (2016) increased the NADPH concentration by overexpressing *Zwf* gene (encoding glucose-6-phosphate dehydrogenase) in *Synechocystis* sp. PCC 6803 and observed an enhancement in ethanol production up to 0.59 g/L under autotrophic condition. Apart from pyruvate to ethanol conversion steps, NADPH is also reported to improve the Calvin cycle, TCA cycle, and acetyl CoA formation through oxidoreduction reactions in *Synechocystis* sp. PCC 6803 (Hasunuma et al., 2014); thereby improving the ethanol production. However, as the NADPH dependent alcohol dehydrogenase gene is heterologously expressed in *S. elongatus*, the demand for NADPH might be increased upon the insertion of a particular gene. Obviously, the experimental results also showed that the NADP^+^ addition increases ethanol production to 1345 mg/L (**Figure 7**), which is higher than that of the NADPH engineered strain (0.59 g/L) (Choi and Park, 2016). In conclusion, the cofactors TPP and NADP^+^ are necessary factors for the improvement of ethanol synthesis in engineered *S. elongatus*. The extracellular addition of such cofactors are not economically feasible; however, the overexpression of particular genes involved in TPP and NADP^+^ synthesis can lead to an improved regulatory mechanism for re-directing the primary carbon sources into ethanol synthesis pathway.

### Pathway Engineering and Cofactor Supplementation Induces Accumulation of Exopolysaccharides

In cyanobacteria, the exopolysaccharides are accumulated under various stress conditions as a putative physical protective mechanism of the cell (DePhilippis and Vincenzini, 1998; Li et al., 2001; Zeyons et al., 2009; Planchon et al., 2013). The present study is focused on exopolysaccharides production in response to engineering of ethanol synthesis pathway and cofactor supplementation. As presented in the results, the insertion of ethanol synthesis pathway improved exopolysaccharides production, which is due to the formation of a cover-shield in response to the stress condition imposed by ethanol synthesis. In addition, the glycogen synthesis pathway destructed strain showed further improvement in exopolysaccharides content, which might be due to the functioning of exopolysaccharides as an alternative to the glycogen. On the other hand, it is also possible that there is the diversion of most of the carbon sources of glycogen pathway to other relatively close pathways, such as xylose, arabinose, and galactose synthesis. The presence of uronic acids and pentoses (xylose, arabinose, and ribose) are the peculiar components of cyanobacteria which makes them negatively charged. This negatively charged characteristic of cyanobacterial surface usually shows a high affinity for metal cations and other positively charged molecules (DePhilippis and Vincenzini, 1998). Additionally, the presence of ribose, fructose, galactosamine, glucosamine, and in some cases, *N*-acetyl glucosamine, 2,3-*O*-methyl rhamnose, and 3-*O*-methyl glucose have been reported (De Philippis et al., 2011).

Irrespective of the strains used, the concentration of sugars were varied in which glucose, galactose, and xylose were in high concentration followed by mannose, arabinose, and uronic acid. Similar pattern was reported for *Anacystis nidulans* by Sangar and Dugan (1972) in which the glucose, galactose, and mannose were in the ratio of 60:14:20. On the other hand, in terms of production, the results of the present study showed lower exopolysaccharides content than that of *Synechocystis* sp. PCC 6803 (36 pg/cell) (Panoff et al., 1998), However, the results showing an increase in exopolysaccharides content upon cofactor supplementation (**Figure 7**) would be beneficial to further stimulate the exopolysaccharides production in *Synechocystis* sp. PCC 6803. The increase in exopolysaccharides along with ethanol concentration clearly indicates that the exopolysaccharides were accumulated in response to ethanol stress condition, which is an added advantage in biorefinery approach.

## Conclusion

An integrative expression of a pyruvate decarboxylase *pdc* from *S. cerevisiae* and an alcohol dehydrogenase *adh* from *Synechocystis* worked well in *S. elongatus*. This *S. elongatus* overexpressing both *adh* and *pdc* showed drastic improvement in ethanol synthesis when its glycogen synthesis pathway was knocked out. The supplementation of co-factors such as Mg^+2^, Zn^+2^, TPP, and NADP^+^ improved further the ethanol production. As a result of engineering, the engineered cells undergo various metabolic changes to cope with the changes in metabolic flux. On the other hand, as a stress induced cellular response, the engineered *S. elongatus* increased the content of exopolysaccharides. Altogether, the engineered *S. elongatus* produced the maximum ethanol yield of 3856 mg/L. In conclusion, the glycogen deficient *S. elongatus* produced exopolysaccharides as a stress response even at a low ethanol concentration. Additionally, the results clearly showed that the improvement of co-factors availability can further promote the ethanol production in *S. elongatus*.

## Data Availability Statement

All datasets generated for this study are included in the article/**Supplementary Material**.

## Author Contributions

RV and AI designed this research and wrote the manuscript. RV performed all experiments.

## Funding

RV is thankful to the Graduate School and Faculty of Science, Chulalongkorn University (CU), for senior post-doctoral fellowship from Rachadaphiseksomphot Endowment Fund. AI acknowledges the research grants from CU on the Frontier Research Energy Cluster (CU-59-048-EN) and from Thailand Research Fund (IRG 5780008).

## Conflict of Interest

The authors declare that the research was conducted in the absence of any commercial or financial relationships that could be construed as a potential conflict of interest.
