# Supplementary material for: Heterologous Expression of Ethanol Synthesis Pathway in Glycogen Deficient Synechococcus elongatus PCC 7942 Resulted in Enhanced Production of Ethanol and Exopolysaccharides
Source: Front Plant Sci. 2020 Feb 14;11:74. doi: 10.3389/fpls.2020.00074 (PMC7034368; doi:10.3389/fpls.2020.00074)
Supplement: Supplementary file 1 [file Image_1.pdf]

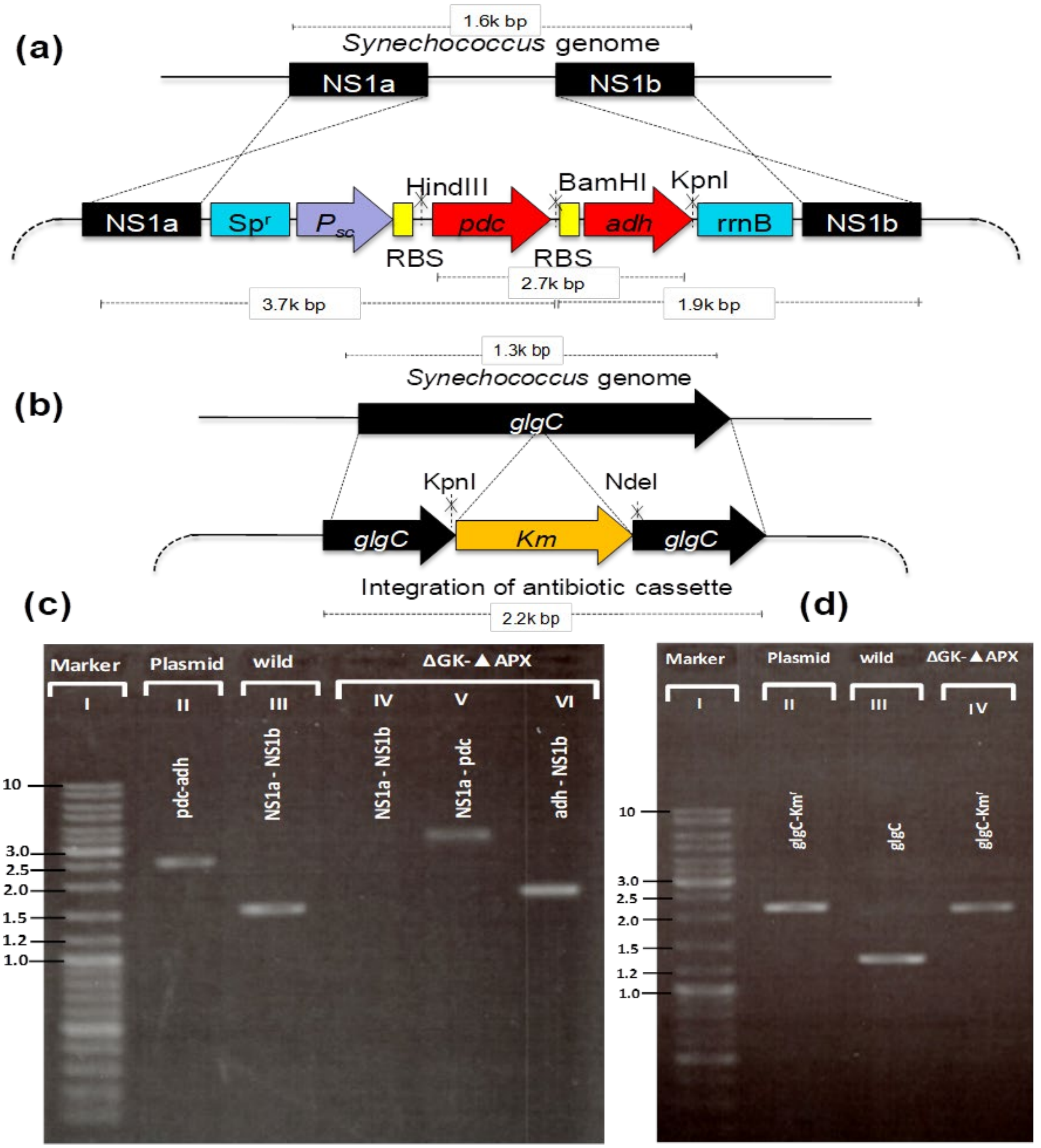

Fig.S1 Scheme of integration of *pdc-adh* cassette into *S. elongatus* PCC 7942. (**a**) Design of *adh-pdc* expression construct. (**b**) Design of *glgC* knockout construct**. (c)** Genotype analysis of ▲APX. Lane I DNA ladder (Fermentas), lane II is the PCR product of *adh-pdc* in plasmid, lane III is the PCR product of *NS1* in wild type. Lane IV, V and VI are the PCR products of *pdc-adh* in engineered strain ΔGK-▲APX. **(d)** Genotype analysis of *glgC* knockout in ΔGK-▲APX. Lane I is the marker, Lane II is the *glgC-Km^r* in plasmid, Lane III is the *glgC* in wild type and Lane IV is the *glgC-km^r* in ΔGK-▲APX. These results indicated the *pdc* and *adh* genes were successfully inserted into the *NS1* site of the genome and *glgC* gene is knocked out by *km^r* cassette.
